# A Proof-of-Concept for a Hypolipidemic Brown Trout Model

**DOI:** 10.3390/toxics12030219

**Published:** 2024-03-15

**Authors:** Tiago Lourenço, Eduardo Rocha, José Fernando Gonçalves, Maria João Rocha, Tânia Vieira Madureira

**Affiliations:** 1 Team of Animal Morphology and Toxicology, Interdisciplinary Centre of Marine and Environmental Research (CIIMAR/CIMAR), University of Porto, Terminal de Cruzeiros do Porto de Leixões, Av. General Norton de Matos s/n, 4450-208 Matosinhos, Portugal; up201707353@edu.icbas.up.pt (T.L.); jfmg@icbas.up.pt (J.F.G.); mjrocha@icbas.up.pt (M.J.R.); 2 Laboratory of Histology and Embryology, Department of Microscopy, ICBAS—School of Medicine and Biomedical Sciences, University of Porto, Rua Jorge Viterbo Ferreira 228, 4050-313 Porto, Portugal; 3 Department of Aquatic Production, ICBAS—School of Medicine and Biomedical Sciences, University of Porto, Rua Jorge Viterbo Ferreira 228, 4050-313 Porto, Portugal

**Keywords:** 17α-ethinylestradiol, atorvastatin, juvenile brown trout, lipids, lipid-lowering drugs

## Abstract

The impacts of hypolipidemic pharmaceuticals on fish lipid metabolism remain unexplored. However, data points to similar effects and mechanisms of action between fish and humans. Therefore, fish may be a strong model for screening hypolipidemic drug candidates and water pollution by lipid-modulating agents. This study aimed to test a new hypolipidemic model assay with juvenile brown trout using atorvastatin (ATV)—a hypolipidemic chemical. We selected 17α-ethinylestradiol (EE2), known to cause hyperlipidemia in fish, to ensure model functionality. Fish received intramuscular injections of 4 μL/g for two weeks under the following experimental conditions: control—C (0.7% NaCl), solvent control—SC (0.7% NaCl, 0.9% ethanol, 0.1% dimethyl sulfoxide), ATV (0.3 μg/g), EE2 (2 μg/g), and a mixture of both compounds—MIX (0.3 μg/g ATV and 2 μg/g EE2). Endpoints included blood lipid biochemistry, hepatic lipid droplet quantification, and liver mRNA expression of lipid-related target genes (related to lipogenesis, lipid transport, and β-oxidation pathways). ATV lowered blood total cholesterol, high-density lipoproteins (HDL), and low-density lipoproteins (LDL) levels, whilst triglycerides and very-low-density lipoproteins (VLDL) were highest under EE2. Hepatic lipid droplet deposition significantly increased in the ATV, EE2, and MIX groups. ATV and MIX caused a significant downregulation of the peroxisome proliferator-activated receptor γ (*pparγ*) and acetyl Co-A oxidase 3 (*acox3*). EE2 upregulated acyl-CoA long-chain synthetase 1 (*acsl1*) and downregulated both fatty acid binding protein 1 (*fabp1*) and acetyl Co-A oxidase 1-3I (*acox1-3I*). ATV caused hypolipidemic effects in juvenile brown trout and could even counteract EE2-stimulated hyperlipidemia, reinforcing the potential of fish hypo- and hyperlipidemic models.

## 1. Introduction

Lipids are key biomolecules in fish, either incorporated in cellular membranes, stored in adipose tissue, and oxidized to produce energy [1] or involved in other processes such as the uptake of very-low-density lipoproteins (VLDLs) to the oocytes for vitellogenesis in fish [2]. Similarities in lipid regulation between fish and humans in numerous metabolic pathways have long been proposed [3]. In both mammals and fish, lipogenesis—the process of de novo synthesis of fatty acids (FAs)—occurs mainly in the liver [1,4], beginning with the oxidation of acetyl-CoA into malonyl-CoA by acetyl-CoA carboxylase (ACC), followed by the assembly of malonyl-CoA by fatty acid synthetase (FAS) in the cytosol [5,6]. The transport of lipids seems also very similar between fish and mammalian models, including the uptake of lipids to the cells, which is mediated by lipoprotein lipase (LPL) [5,7], the intracellular shuttling of FAs performed by fatty acid binding proteins (FABP) [7,8], or the plasmatic transport of lipids and FAs, which is primarily regulated by lipoproteins that contain specific carrier apolipoproteins [6]. FA catabolism is one of the primary energy sources of fish and, as in mammals, it occurs mainly by the β-oxidation of FAs, the cleavage of long-chain FAs at the β-carbon, and takes place in the mitochondria or the peroxisomes [6,9].

There is a defined network of transcription factors that regulate hepatic lipid metabolism, which includes the sterol regulatory element-binding protein (SREBPs), liver receptor X (LXR), retinoid receptor X (RXR), and peroxisome proliferator-activated receptors (PPARs) [10]. Further, lipid and fatty acid metabolism processes are co-regulated by hormones. For instance, in fish, during vitellogenesis, 17β-estradiol (E2) activates estrogen receptors (ERs) in the liver, with the subsequent synthesis of vitellogenin, the precursor of egg yolk proteins, which is later sequestered by the oocytes [11]. Experimental induction of lipid modulation by sex steroids in fish has also been studied. For example, a 30-day exposure of female triploid rainbow trout (*Oncorhynchus mykiss*) to testosterone (T) and E2-treated diets showed that both sex steroids downregulated *acc* mRNA expression and E2 alone decreased *lpl* and *pparγ* mRNA levels [12], whilst the waterborne exposure of brown trout (*Salmo trutta fario*) to 17α-ethinylestradiol (EE2) at 50 µL/L upregulated *pparα* and acyl-CoA long-chain synthetase 1 (*acsl1*) and downregulated *pparγ*, acyl-CoA oxidase 1-3I (*acox1-3I*), *acox3*, *fabp1*, and apolipoprotein AI (*apoa1*) mRNA levels in the liver [13].

Dyslipidemias are pathologies that can encompass a wide range of blood lipid abnormalities, often resulting in excessively high levels of cholesterol and triglycerides (TGLs) [14]. To overcome coronary heart disease conditions associated with dyslipidemia, lipid-lowering agents, such as statins, have been increasingly used, particularly in high-income regions like Europe [15], where their low price makes them readily available, unlike in low-income regions [16]. Atorvastatin, simvastatin, and rosuvastatin are the most prescribed within the statin family, with atorvastatin (ATV) being one of the most prescribed pharmaceuticals worldwide [17]. Statins lower blood cholesterol levels by inhibiting the activity of hydroxy-3-methylglutaryl coenzyme A reductase (HMGCOAR) [18]. The HMGCOAR enzyme catalyzes the rate-limiting step of cholesterol biosynthesis via the mevalonate pathway [19], which is highly preserved across vertebrate taxa, as is the structure of the enzyme [20]. In fact, there is evidence that statins can lower cholesterol levels and interfere with the expression of *hmgcoar* in fish [21,22], similar to what has been demonstrated in humans. Additionally, to the importance of animal testing for screening new lipid-lowering candidates [23], water pollution by established drugs is an emerging reality, with poorly understood consequences for aquatic biota, particularly for fish species [24].

The primary goal of this study was to develop a fish hypolipidemic testing model using juvenile brown trout, which could be an effective controlled biomedical and ecotoxicological tool due to the conservation of lipid pathways between humans and fish. ATV was used to induce hypolipidemia against a selected hyperlipidemic control. EE2 is one of the most popular synthetic estrogens commonly used in oral contraceptives, which have a recognizable capacity to disrupt lipidic pathways in fish, namely causing hyperlipidemic responses such as increasing circulating TGL and VLDL levels in brown trout [13] and hybrid tilapia [25]. EE2 and ATV interfere in two closely related liver pathways, vitellogenesis and cholesterol biosynthesis, respectively, in the sense that both imply changes in lipid handling and metabolism. A mixture of both compounds was also tested to validate the capability of ATV to counteract the lipid-raising effects of EE2, but this study did not set out to disclose the mechanistic interconnection between pathways. Thus, we hypothesized that ATV could generate a brown trout hypolipidemic model and that under additional stimulation with EE2, it reversed (at least partially) the ATV effects. The characterization of the lipidic changes enacted by ATV was measured across several endpoints that included blood lipid biochemistry, deposition of lipids in the liver, and alterations in the hepatic expression of genes involved in lipogenesis, cholesterol metabolism, lipid transport, and peroxisomal β-oxidation.

## 2. Materials and Methods

### 2.1. Animals

Immature juvenile (1-year-old) brown trout were obtained from a government rearing facility for repopulation (Torno, Amarante, Portugal) and maintained for 28 days before experiments, as recommended for this species [26]. Batches of 10 fish were randomly allocated in 100 L fiberglass tanks and kept in an artificial photoperiod of 12 h of light and 12 h of dark. The fish were fed daily (Trout Plus 4, AquaSoja, Ovar, Portugal) except for the day before handling (injections and sampling). Half the water volume in each tank was renewed with dechlorinated water every other day. Water temperature (16.6 ± 0.4 °C) and dissolved oxygen levels (89.5 ± 3.8%) were measured daily, before and during the experiment, using a portable instrument (DO210, VWR International, Leuven, Belgium). Levels of nitrite (NO_2_^−^—0.61 ± 0.5 mg/L), ammonia (NH_3_—0.31 ± 0.4 mg/L), ammonium (NH_4_—0.31 ± 0.4 mg/L), and pH (7.9 ± 0.6) were monitored twice a week with commercial test kits for NH_3_/NH_4_ and for NO_2_^-^ (Prodac, Cittadella, Italy), and a pH reader (WTW pH530, Oberbayern, Germany).

All fish handling and assay procedures were performed in accordance with the Portuguese Decree-Laws No. 113/2013 and No. 1/2019 and the guidelines from the 2010/63/EU European Directive and supervised by experts accredited by the Portuguese Directorate-General for Food and Veterinary in laboratory animal science following FELASA category C recommendations.

### 2.2. Exposure Procedures

Five independent tanks (9 fish each) were set up, and each one was randomly assigned to one of five experimental conditions: (1) control (C), which corresponded to 0.7% saline solution, by diluting 0.7 g of NaCl (VWR International, Leuven, Belgium) in 100 mL of autoclaved distilled water; (2) solvent control (SC), consisting of the previous saline solution fortified with dimethyl sulfoxide (DMSO, VWR Chemicals, Solon, OH, USA) and ethanol (Merck KGaA, Darmstadt, Germany) at 0.1 and 0.9%, respectively; (3) 17α-ethinylestradiol (EE2, Sigma-Aldrich, St. Louis, MO, USA; CAS: 57-63-6) at 2 μg/g of body weight prepared in SC solution; (4) atorvastatin (ATV, LGC Ltd., Teddington, UK; CAS: 134523-03-8) at 0.3 μg/g of body weight in SC solution; and (5) a mixture (MIX) of EE2 (2 μg/g of body weight) and ATV (0.3 μg/g of body weight). The SC solution was used to ensure the solubility of both ATV and EE2. ATV concentration was defined according to the solubility, and the levels tested in a previous in vivo zebrafish (*Danio rerio*) exposure to ATV at 0.53 μg/g of body weight used to replicate human pharmacological doses of this statin, which range from 0.14 to 1.14 μg/g of body weight [21]. EE2 concentration was selected based on previous exposures of rainbow trout (*Oncorhynchus mykiss*) to 17β-estradiol (E2) and two phytoestrogens (genistein and daidzen), at 5 μg/g of body weight via injection that produced effects in estrogenic and lipidic targets [27].

The experimental solutions were administered via intramuscular injection every third day for 14 days (making up 4 injections per fish). This injection periodicity was previously used to expose rainbow trout to other hypolipidemic pharmaceuticals via intraperitoneal (IP) injection and induce effects on lipidic targets [28]. Further, Mimeault et al. [29] showed that the hypolipidemic fibrate gemfibrozil (GEM) was about 95% eliminated from the plasma of goldfish (*Carassius auratus*) within 96 h after intraperitoneal injection. Injections every third day were also used to expose male platyfish (*Xiphophorus maculatus*) to EE2 via intramuscular injection, increasing the vitellogenin levels in the liver, without physical harm to fish [30]. Injection solutions were freshly prepared before every exposure and administered at 4 μL/g of body weight, which is within the range of injection volumes used with Atlantic salmon (*Salmo salar*) [31], and rainbow trout [27]. Fish were anesthetized individually with 250 μL/L of glycol monophenyl ether (Merk KGaA, Darmstadt, Germany) until they reached stage III, plane 3 of deep narcosis [32]. Injections were always made above the lateral line and immediately behind the dorsal fin, alternating sides at each consecutive injection. After the injection, the fish were maintained in a recovery tank with highly oxygenated water and under similar conditions to the housing tank until they regained balance and resumed swimming behavior. Finally, the fish were returned to the housing tank after recovering from the injection procedure.

### 2.3. Sampling

Fish were euthanized with an overdose of ethylene glycol monophenyl ether (1 mL/L), weighted, and measured for total length. Total blood was immediately collected from the caudal vein with 1 mL syringes (BD Plastipak^TM^, Becton Dickinson, Vaud, Switzerland) and placed into K3-EDTA coated tubes (Vacuette^®^, Greiner Bio-One, Kremsmünster, Austria). The liver was removed and weighted before being sectioned into 2-mm-thick fragments and either fixated in neutral buffered formalin at 10% (Epredia, Breda, The Netherlands) for histological analysis or snap-frozen in liquid nitrogen and stored at −80 °C for molecular analysis.

Hepatosomatic index (HSI) and Fulton’s condition factor (K) were calculated according to the following formulas: HSI = 100 × liver weight (g)/fish weight (g); K = 100 × fish weight (g)/(fish total length)^3^ (cm).

### 2.4. Blood Lipid Biochemistry

Blood lipid levels were measured using the Cobas b101 analyzer (Roche Diagnostics, Rotkreuz, Switzerland). For each fish, a 20 μL sample of K3-EDTA anticoagulated whole blood was loaded into a Lipid Panel Test disc (Roche Diagnostics, Rotkreuz, Switzerland), which automatically determines total cholesterol, high-density lipoproteins (HDL), and triglycerides (TGLs). Low-density lipoprotein (LDL) levels were determined as follows: LDL = total cholesterol − HDL − (TGLs/5). VLDL concentration was calculated using the formula: VLDL = TGLs/5 [33]. Non-HDL cholesterol was calculated by subtracting HDL concentration from total cholesterol. The HDL/LDL, LDL/HDL, TGLs/HDL, and total cholesterol/HDL ratios were also determined.

### 2.5. Liver Lipid Quantification

The lipid droplets in the liver were quantified in one 2 mm fragment, representing 16% of the total liver, fixated in neutral buffered formalin at 10% (Epredia, Breda, The Netherlands) for 24 h. The fragment was post-fixated in a 2% osmium tetroxide solution (Agar Scientific, Stansted, Essex, UK) buffered in 0.2 M cacodylate buffer (Merck, Darmstadt, Germany) and potassium dichromate at 5% (BDH Chemicals Ltd., Poole, UK), following a protocol described for lipid fixation and staining in mice with few adaptations for the rinsing periods [34]. The liver fragments were submerged in the osmicating solution and agitated for 8 h, followed by a 1 h period of repetitive 1 min rinses in water. After, the osmicated liver fragments were processed in an automated tissue processor (TP1020, Leica Biosystems, Wetzlar, Germany) and embedded in paraffin (Histoplast, Epredia, Vreda, The Netherlands) using an embedding station (EG1140C, Leica Biosystems, Wetzlar, Germany). Paraffin blocks were sectioned in an automated rotary microtome (RM2255, Leica Biosystems, Wetzlar, Germany) at 3 μm from random points in the block. Two sections per animal were mounted on silane-coated microscope slides (VWR International, Leuven, Belgium) and left to dry for 48 h at 37 °C. The slides were then coverslipped with mounting medium (DPX, Sigma-Aldrich, St. Louis, MO, USA) without staining. This was done to enhance the contrast between the black-stained lipid droplets and the rest of the tissue.

A total of 12 systematically sampled fields of liver parenchyma/fish were photographed under a light microscope (Olympus BX50, Tokyo, Japan) equipped with a camera system (Olympus EP50, Tokyo, Japan) using the 40× objective. Lipid droplet content was quantified in the photographs using the Fiji ImageJ software (version 1.53). The images were converted to black-and-white 32-bit files, and a threshold was applied to the black-stained lipid droplets. The software quantified the marked black areas as a percentage of the total image area. The quantification result per fish was the average of the 12 fields. The relative area of the lipid droplets in all the randomly sampled fields was used to estimate the relative volumes of the lipid droplets in the liver [35]: V_v_ (lipid droplets, liver parenchyma).

### 2.6. Liver RNA Expression

Total RNA extraction was made from a 14 to 20 mg homogenized liver fragment using the illustra^TM^ RNAspin Mini Isolation Kit (GE Healthcare, Chicago, IL, USA). The kit included an in-column DNAse I digestion step. The obtained RNA samples were quantified using a microplate spectrophotometer Multiskan Go (Thermo Scientific, Vantaa, Finland) running with the SkanIt software (version 4.1, Thermo Fischer Scientific, Waltham, MA, USA). The optical density ratios at 260/280 nm and 260/230 nm, along with 1% agarose gel electrophoresis stained with GelRed (Biotium, Fremont, CA, USA), were used for evaluating RNA sample integrity. cDNA was synthesized from 1 μg of total RNA using the iScript cDNA synthesis kit (BioRad, Hercules, CA, USA), for a total volume of 20 μL.

Expression of molecular targets (Table 1) in the liver was done by quantitative real-time polymerase chain reaction (qRT-PCR) using a Real-Time PCR Detection System (CFX Connect, BioRad, Hercules, CA, USA). Each qRT-PCR reaction had a total volume of 20 μL that included 5 μL of the cDNA sample diluted 1:5, 10 μL of either iQ^TM^ SYBR^®^ Green Supermix (BioRad, Hercules, CA, USA) or SsoFastTM EvaGreen^®^ Supermix (BioRad, Hercules, CA, USA) and 200 nM or 300 nM of the primers for the specific target, respectively. The acyl-CoA oxidase 1-3I (*acox1-3I*), acyl-CoA oxidase 3 (*acox3*), and peroxisome proliferator-activated receptor gamma (*pparγ*) were optimized with the SsoFastTM EvaGreen^®^ Supermix, whilst the remaining targets (Table 1) were optimized with iQ^TM^ SYBR^®^ Green Supermix. All plates had duplicate samples and contained no template controls (NTC). A melting curve from 55 to 95 °C with 0.5 °C increments was generated in each run to ensure product specificity. The expression of the target genes was normalized according to the Pfaffl method [36], using a multiple reference gene approach [37]. The geometric mean of glyceraldehyde-3-phosphate dehydrogenase (*gapdh*) and ribosomal protein L8 (*rpl8*) was used for the normalization because they were the most stable gene combination given by NormFinder algorithm from a selection of four genes (*rpl8*, *gapdh*, beta-actin—*β-actin*, and elongation factor 1α—*ef1 α*) [38].

### 2.7. Statistical Analysis

The statistics were performed with the PAST 4.3 software [48]. Biometric parameters, blood lipids, hepatic lipid quantification, and gene expression were compared between groups with one-way analysis of variance (ANOVA), followed by Tukey’s pairwise post-hoc test. ANOVA assumptions of homogeneity of variances and normality were checked using Levene’s test and the Shapiro–Wilk test, respectively. Data failing the premises were analyzed with the non-parametric Kruskal–Wallis H test followed by the Mann–Whitney U post-hoc test with sequential Bonferroni correction. Significant differences were considered when *p* < 0.05.

## 3. Results

### 3.1. Mortality and Fish Biometry

During the 14-day injection assay, there was no mortality at all. Fish biometry is summarized in Table 2. No significant differences were observed for the total weight, total length, and the condition factor (K) across the experimental groups. During the assay, there were no major differences in the weights of the animals in each group (Figure S1). The liver weight was significantly higher in the EE2 group compared with the remaining groups. This extra weight in EE2-treated fish liver was accompanied by a softer consistency and yellowish color of the organ. As a result, the HSI of EE2-exposed fish was also significantly higher when compared to all other groups. A significant increase in HSI was also noted in the MIX group compared to the controls and ATV group.

### 3.2. Blood Lipid Biochemistry

The blood lipid levels for all experimental groups are summarized in Table 3. In the C group, the median values of total cholesterol, LDL, and non-HDL cholesterol were 307 mg/dL, 159.8 mg/dL, and 203 mg/dL, respectively. Those levels decreased significantly in the ATV group compared to all other groups (Table 3). The HDL median levels were significantly lower in EE2 (37.5 mg/dL), ATV (101 mg/dL), and MIX (93 mg/dL) groups in contrast to both controls (C and SC—105 mg/dL). TGL and VLDL levels followed a very similar pattern, with both being significantly higher in the EE2 group (TGLs—651 mg/dL; VLDL—130.2 mg/dL) and the lowest in the ATV group (TGLs—181 mg/dL; VLDL—36.2 mg/dL) in comparison to the controls (Table 3). The total cholesterol/HDL and LDL/HDL ratios were also significantly increased in the EE2 group and decreased in the ATV group compared to controls. On the contrary, the HDL/LDL ratio decreased significantly under EE2 treatment and increased with ATV, whereas the TGLs/HDL ratio was significantly increased in the EE2 group and reduced in the MIX.

### 3.3. Liver Lipid Quantification

**Figure 1:**
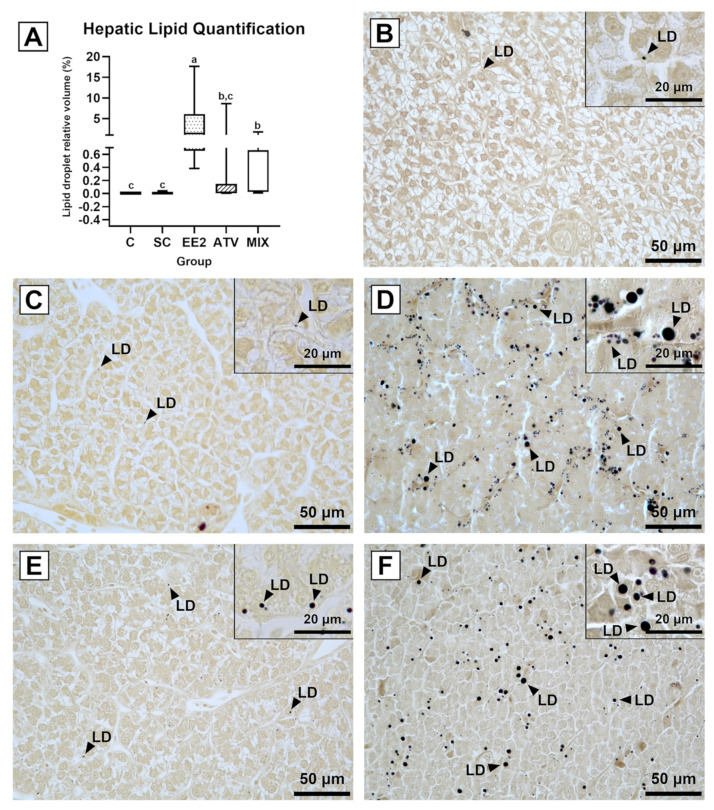
Lipid droplet content in juvenile brown trout liver. Relative volumes of lipid droplets—V_v_ (lipid droplets, liver parenchyma) (**A**); data are expressed as minimum, maximum, and median percentage values for each group with *n* = 9 animals/group. Different lower-case letters indicate significant differences (*p* < 0.05) between groups. Representative histological sections of osmicated liver from the control (**B**), solvent control (**C**), 17α-ethinylestradiol (**D**), atorvastatin (**E**), and mixture (**F**) groups. The sections evidenced lipid droplets (LD) as dark deposits in liver parenchyma.

The relative volume of the lipid droplets in the C and SC ranged from 0.003% to 0.007% and 0.003% and 0.042%, respectively (Figure 1A). EE2 and MIX caused a significant increase in the deposition of lipid droplets in the liver parenchyma, contrasting with both controls (Figure 1A). The EE2 group had the highest increase in the % of lipid content, ranging from 0.383% to 17.640%. Lastly, the droplets’ volume density in the ATV and MIX groups was between 0.004% to 8.610% and 0.010% to 1.729%, respectively, and was significantly lower than in the EE2 group (Figure 1A).

Qualitative analyses from the histological sections agreed with the quantitative changes in lipid deposition (Figure 1B–F). The control groups had nearly absent tiny lipid droplets (Figure 1B,C). In the EE2 group, the droplets were the largest observed, abundant, and homogeneously distributed (Figure 1D). The droplets in the ATV group showed an apparent reduction in size when compared to EE2 and were sparser and distributed in clusters (Figure 1E). The MIX liver parenchyma had droplets with a size more compatible with those found in the EE2 group, but they were distributed in less abundant clusters, as in the ATV treatment (Figure 1F).

### 3.4. Liver RNA Expression

**Figure 2:**
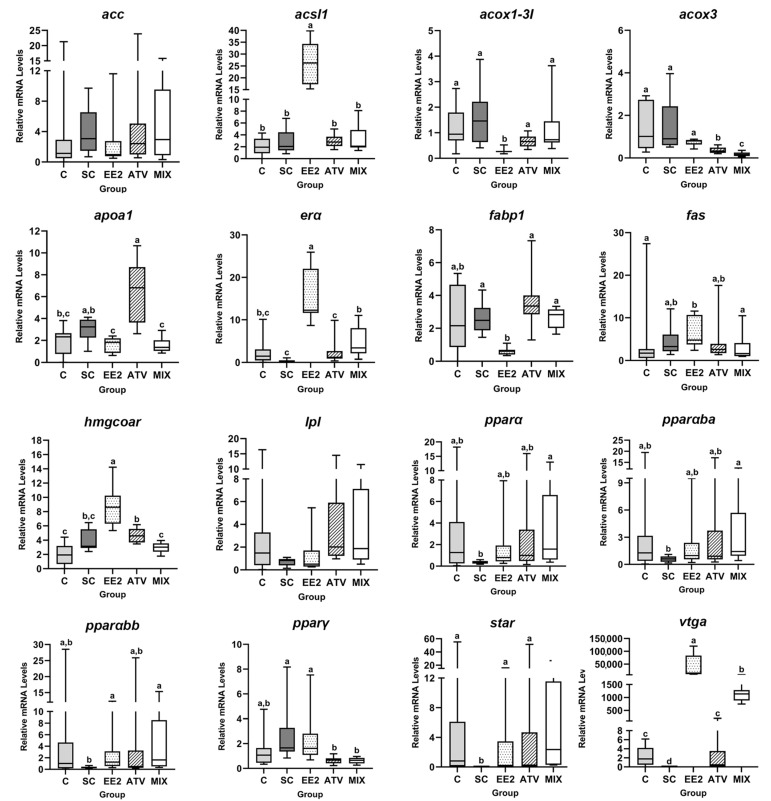
Liver relative mRNA levels of the distinct target genes in juvenile brown trout exposed to the following: C—control, SC—solvent control, EE2—17α-ethinylestradiol, ATV—atorvastatin, and MIX—mixture. Data are expressed as median, minimum, maximum, and the 25th and 75th percentiles for *n* = 9 fish/group. Different lower-case letters indicate significant differences (*p* < 0.05) between groups.

The mRNA levels of the distinct liver target genes are shown in Figure 2. The estrogenic targets (*vtga* and *erα*) were significantly upregulated in the EE2 group relative to the controls (over 35,000-fold for *vtga* and 10.8-fold for *erα*) and ATV and MIX groups. The *acsl1* and *hmgcoar* mRNA levels were also significantly upregulated in the EE2 group (a 10-fold increase for *acsl1* and 8.7-fold for *hmgcoar*) compared to all other conditions. Those increases caused by EE2 were both reverted in the MIX group. The *hmgcoar* mRNA levels were also significantly upregulated (1.5-fold increase) in the ATV group versus the C group. The *fabp1* mRNA levels were significantly downregulated by EE2 (4.4-fold decrease) compared to all other groups except for the C group. The *apoa1* expression was also downregulated in the EE2 and MIX groups, contrary to the ATV group (a 1.5-fold decrease for EE2 and MIX and a 2.6-fold increase for ATV). No major changes were noted in the mRNA levels of *acc*, *lpl*, *fas*, and *star*.

The mRNA levels of *acox1-3I* were significantly lowered by EE2 (5-fold decrease) regarding the remaining groups. The *acox3* mRNA levels were significantly reduced in ATV and MIX compared to controls and EE2 groups (3.9-fold decrease for ATV and 8-fold decrease for MIX). A similar profile was found for *pparγ* expression levels (3-fold decrease for both ATV and MIX). Lastly, *pparα* mRNA levels and its isoforms—*pparαba* and *pparαbb*—remained mostly unchanged in response to EE2, ATV, and MIX.

## 4. Discussion

**Figure 3:**
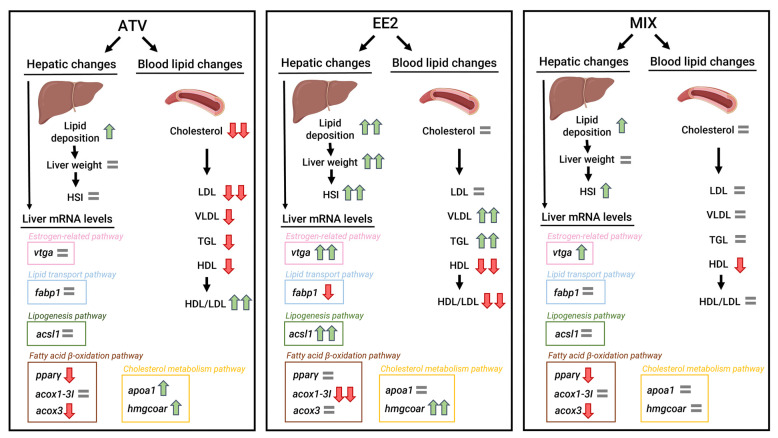
Simplified summary of the changes caused by the ATV, EE2, and MIX treatments. One colored arrow means a statistically significant increase (green upward arrows) or decrease (red downward arrows) relative to the control, while two colored arrows indicate a statistically significant increase (green) or decrease (red) relative to all experimental groups. The grey equal sign means there is no significant change relative to the control.

Despite the relevance of establishing new alternative/additional models for testing hypolipidemic pharmaceuticals, such as statins and fibrates [49], a limited number of studies have evaluated the lipidic effects caused by hypolipidemic compounds in fish. This study attempted to establish a new fish model for hypolipidemia, using juvenile brown trout as a model organism due to its similarities to mammals in lipid metabolism [3]. A positive control of hyperlipidemia was also included using EE2 to prove our model’s functionality. EE2 caused an excepted increase in *erα* and *vtga* mRNA expression in the liver. Both estrogenic effects were previously demonstrated in vitro and in vivo in brown trout hepatocytes after EE2 exposures [13,42,45]. A simplified graphical summary of the effects caused by each treatment and a comparison between them is shown in Figure 3.

The blood lipid profiles in the C and SC groups were similar to those reported for the same species [13] and the Caspian brown trout [50]. ATV exposure decreased total cholesterol levels, aligning with the cholesterol-lowering effects observed in zebrafish treated with ATV [21]. Similarly, fibrates administered to rainbow trout [28] and grass carp (*Ctenopharyngodon idellal*) [51] also exhibited a reduction in total cholesterol levels. Resembling what has been observed in fish, ATV in humans inhibits cholesterol biosynthesis by stopping the mevalonate pathway, causing a drop in cholesterol levels [14,18]. In this study, HDL levels were lower in all treatment groups. Although the ATV treatment resulted in a drop in HDL, the HDL/LDL ratio showed that the fraction of HDL in these animals’ blood was significantly higher compared to both controls, aligning the effect of ATV in brown trout with the mode of action that has been described in humans [18].

In this study, all non-HDL lipoproteins were lowered after exposure to ATV. LDL levels were markedly reduced in fish that underwent ATV treatment compared to all other treatments. This reduction was comparable to the effect of fibrate exposure observed in rainbow trout [28] and grass carp [51]. In the human liver, ATV is expected to trigger an increased abundance of LDL receptors (LDLRs) [52] concomitantly with increased expression of HMGCoAR after cholesterol depletion, which is consistent with a common transcriptional regulation [53]. Increased expression of *ldlr* in rainbow trout hepatocytes has been shown after in vitro exposure to ATV [54], and it could be a mechanism leading to liver uptake of LDL from the blood and lowering of its plasmatic concentration. ATV also reduced VLDL levels, a similar outcome seen in rainbow trout exposed to another hypolipidemic drug (gemfibrozil) [28]. Statins have also been proposed to disrupt the assembly of VLDLs, thus lowering their concentration in the blood [52]. Along with the decrease in VLDLs, the TGL levels were also reduced in the ATV-treated brown trout, as reported in female zebrafish exposed to the same statin and to gemfibrozil [21]. By contrast, EE2 elicited a hyperlipidemic response, significantly increasing VLDL and TGL blood concentrations. Juvenile brown trout exposed through the water to EE2 at 50 μg/L also showed higher circulating levels of TGLs and VLDLs [13]. The rising levels of VLDLs and TGLs in our experiment under EE2 influence can be due to two phenomena: (a) EE2 promotes TGL transport to the liver during the process of EE2-induced vitellogenesis [2]; (b) whilst in the bloodstream, TGLs are mainly transported by VLDLs [55].

In the present study, both control groups had tiny and scarce lipid droplets after the staining with osmium tetroxide, which agrees with the lipid droplet descriptions in juvenile Atlantic salmon hepatocytes [56]. In contrast, the livers of EE2-exposed brown trout had the highest amount of lipid droplet deposition, like what occurred after waterborne exposure of juvenile brown trout to EE2 at 50 μg/L [13]. ATV exposure also caused an increase in lipid droplets in the hepatocytes, but not as extensive as the one described after estrogenic inputs. The mechanism of lipid uptake into the liver from the bloodstream could be a consequence of ATV treatment, which is expected to induce *ldlr* expression [54]. As for the MIX group, the amount of lipidic deposition in the liver was significantly lower than that of the EE2 group, but still greater than that observed in the ATV-treated fish.

In line with the more significant lipid droplet content, the livers of fish treated with EE2 and MIX were heavier than those of any other experimental group. Additionally, these livers had a paler color and softer consistency. The animals in those two groups also showed increased HSI. Likewise, increased liver mass and HSI were observed in juvenile brown trout after in vivo waterborne exposure to EE2 [13]. Still, similar effects registered in the MIX group suggest that EE2 modulates liver biometry more strongly than ATV, at least for the tested concentrations. Regarding HSI, ATV once more acted in a way that counteracts the effect of EE2.

ACC, the enzyme responsible for kickstarting lipogenesis by converting acetyl-CoA into malonyl-CoA [57], did not show herein significant alterations in its hepatic expression in any of the experimental groups. Exposure of male Sprague–Dawley rats to ATV at 0.1% *w*/*w* in the diet for three days caused an upregulation of ACC expression [58], whereas dietary exposure of grass carp to clofibrate at 1.25 g/kg *w*/*w* caused downregulation of the *acc* mRNA levels [51]. Overall, the data from the present work and earlier ones suggest that hypolipidemic pharmaceuticals affect ACC expression differently depending on the exposure conditions and species. Previous reports of *acc* response to E2 in rainbow trout are also not consensual. Intraperitoneal injections of E2 at 5 μg/g of body weight were not able to significantly alter the expression of *acc* [27], whereas a one-month in vivo diet exposure to E2 at a 30 mg/kg diet caused a downregulation of *acc* mRNA levels [12]. The lack of significant alterations to the *acc* expression observed herein suggests that EE2 does not target this enzyme’s expression in the juvenile brown trout liver.

The next step of lipogenesis is catalyzed by *fas* by converting malonyl-CoA into the final product of lipogenesis [6]. In our study, *fas* was not significantly altered by ATV, despite hypolipidemic pharmaceuticals being expected to reduce the activity of *fas* and thus decrease the synthesis of FAs, as observed in grass carp fed with a clofibrate diet [51]. Despite its hyperlipidemic capabilities, EE2 did not upregulate the expression of *fas* in our assay. Also, in rainbow trout and Nile tilapia (*Oreochromis niloticus*), exposure to estrogenic compounds did not cause significant alterations in *fas* liver expression [12,59]. For lipogenesis, FAs must be converted into fatty acyl thioesters by ACSL1 and incorporated into the metabolic pathways [60]. The mRNA levels of *acsl1* were unaltered by ATV treatment. The same occurred in brown trout juveniles treated with clofibrate at 250 µg/L for 28 days [13]. However, hamsters treated by oral gavage with rosuvastatin (20 mg/kg) for seven days showed a significant upregulation of hepatic ACSL1 mRNA [61]. This indicates that the effect of statins on ACSL1 expression differs between mammals and fish but points to a similar response in brown trout liver to distinct hypolipidemic chemicals. The EE2 exposure upregulated *acsl1* mRNA levels, as previously demonstrated in brown trout juveniles exposed to the same compound [13]. This suggests that free fatty acids are being converted into acetyl-CoA and thus used for lipid synthesis in the liver, a rationale in line with the observed increased load of lipid droplets.

Lipoprotein lipase (*lpl*) is responsible for the extracellular uptake of lipids by hydrolyzing plasma TGLs transported through VLDL and chylomicron [6]. Previous in vivo exposures of fish models to two fibrates—gemfibrozil [28,62] and clofibrate [51]—caused upregulation of *lpl* levels as a measure to reduce circulating lipids, but in our study, no changes were noted. This suggests that in brown trout, the mechanism of lipid removal from the bloodstream by statins may not be mediated by *lpl*, but by other membrane enzymes. However, the assessment of the enzymatic activity of *lpl* should be explored to shed further light on this mechanism. The expression of *fabp1*, an intracellular FA shuttler [57,63], was not significantly altered herein by ATV. Contrarily, in zebrafish, the clofibrate administered through diet upregulated the *fabp7a*, *fabp10a*, and *fabp11a* expression in the liver [64]. Also, liver FABP mRNA in primary rat hepatocytes was significantly upregulated after simvastatin treatment (12 and 24 µM) [65]. EE2 treatment downregulated *fabp1* mRNA levels in the present study, which agrees with previous results [13]. This downregulation caused by the hyperlipidemic treatment with EE2 points to a reduction in FA movement within the cytosol to be incorporated into anabolic and catabolic pathways, whereas the lack of significant alterations caused by ATV indicates that ATV do not act by modulating *fabp1* expression, at least under these experimental conditions.

Statins act as competitive inhibitors of HMGCOAR, and Estey et al. [66] have demonstrated that intraperitoneal injection of rainbow trout with cerivastatin at 1.4 ng/g fish can significantly reduce the *hmgcoar-1* mRNA expression after 24 h. However, studies with zebrafish and mice reported higher *hmgcoar* mRNA levels after ATV exposures [21,58], which our data corroborates. An explanation for this phenomenon was attempted by Al-Habsi et al. [21], who suggested that cholesterol reduction led to an upregulation of *hmgcoar* mRNA in an attempt to increase cholesterol biosynthesis and restore original levels. Interestingly, EE2 caused an increase in *hmgcoar* expression in our assay; although, to our knowledge, there is no evidence of estrogenic modulation of *hmgcoar* expression in fish.

APOA1 is the major apolipoprotein in HDL, making up approximately 70% of its apolipoprotein content [67]. It is involved in cholesterol metabolism, mainly through reverse cholesterol transport [67], which results in the selective cholesterol uptake into the liver [68]. Here, *apoa1* mRNA was significantly upregulated by ATV, similar to what gemfibrozil did in seabream (*Sparus aurata*) after waterborne exposure to 150 μg/L [62], as well as what was shown in human HepG2 cells treated with statins [69,70]. An upregulation of *apoa1* mRNA levels would be tied to an increase in reverse cholesterol transport [67], and subsequent liver uptake of HDL. Indeed, the current study shows that HDL concentrations in the blood decreased after ATV treatment. However, despite a decrease in absolute concentrations of HDL, it exhibited a relative increase in relation to the other lipoprotein classes, in accordance with the drug’s mechanism of action in humans [18].

PPARα, a nuclear receptor predominantly found in the liver, plays a crucial role in regulating several aspects of lipid and FA metabolism, namely peroxisomal β-oxidation of FAs [1]. Herein, no treatment significantly affected *pparα* mRNA levels or its two isoforms in brown trout (*pparαba* and *pparαbb*) [41]. However, waterborne exposure of zebrafish embryos to simvastatin at 50 μg/L did cause an upregulation of *pparα* [71], and EE2 at 50 µg/L upregulated *pparα* and *pparαba* in juvenile brown trout [13]. Here, the lack of change to *pparα* and its isoforms could provide a reasonable explanation for the absence of effects produced by ATV on *acsl1*, *fabp1*, and *lpl* expressions, as these genes are regulated by PPARα [72]. As an example, previously, statin therapy was shown to directly regulate liver FABP in mice via PPARα [65].

ACOX1-3I is a direct target of PPARα and catalyzes the first step of peroxisomal FA β-oxidation [72]. We found no data on the influence of statins on the *acox1-3I* mRNA in fish. However, studies on clofibric acid exposure in common carp (*Cyprinus carpio*) via water [73] and male zebrafish (F1 generation) through food [74] showed an upregulation of this gene. In this study, ATV did not cause significant changes, which suggests that statins do not promote FA catabolism via peroxisomal β-oxidation and it is in line with the absence of *pparα* stimulus. EE2 downregulated *acox1-3I* mRNA, which is similar to what happened previously in juvenile brown trout under a 28-day waterborne exposure [13]. ACOX3 is an enzyme also involved in FA catabolism, catalyzing the first step of an alternative pathway of peroxisomal β-oxidation, as shown in humans [75]. Interestingly, *acox3* was downregulated herein by ATV and MIX in a pattern very closely related to that of *pparγ*. This same pattern of downregulation of both *pparγ* and *acox3* was observed in juvenile brown trout exposed to clofibrate [13], which again suggests that statins do not promote FA catabolism and points to the production of similar effects of statins and fibrates in fish, perhaps even with other hypolipidemic pharmaceuticals.

PPARγ is also a major regulator of lipid metabolism, namely adipogenesis and lipid storage [1], as well as peroxisomal β-oxidation [76]. The downregulation of *pparγ* mRNA levels by ATV contradicted results with zebrafish, namely the dietary exposure to ATV at 53 μg/g of food [21] or the waterborne exposure to simvastatin at 50 μg/L [71], both of which upregulated *pparγ*. However, it was in line with the treatment of mice with ATV via the diet that downregulated PPARγ [77] and is closely related to the expression pattern of *acox3*. These close patterns suggest that the catabolic pathway initiated by *acox3* is more strongly induced by *pparγ* rather than *pparα* in the liver of brown trout.

## 5. Conclusions

Juvenile brown trout exposed to ATV via intramuscular injection showed lipid-metabolism-related alterations consistent with hypolipidemic responses elicited by ATV in mammals and other fish models, such as a decrease in circulating lipid levels, increase in hepatic lipid deposition, and changes in the expression of specific target genes (e.g., *pparγ* and *acox3*). Mix response patterns also showed that ATV could reverse the hyperlipidemic effects of EE2. The present outcomes evidence that the proposed juvenile brown trout hyperlipidemic versus hypolipidemic models are a handy and promising experimental tool for testing known or candidate lipid-regulating (or suspected lipid-disrupting) compounds in the presence or absence of a hyperlipidemic context. It should be stressed that this model cannot be linearly used in an environmental context because several aspects should be taken into consideration, namely the exposure concentration and route, and the drug bioavailability and bioaccumulation. Besides the tested endpoints in this study, enzyme activities as phenotypic anchors of gene expression would be significant in future investigations.

## Figures and Tables

**Table 1:** Gene list used for qRT-PCR, with primer sequences, annealing temperatures, and amplification efficiencies.

Gene Name	Abbreviation	Primer Forward (5′-3′)	Primer Reverse (5′-3′)	Annealing Temperature (ᵒC)	Amplification Efficiency (%)	Reference
Acetyl-CoA carboxylase	*acc*	TTTTGATGGCGATCTTGACA	CATCACAATGCCTCGCTCTA	60.0	102.2 ^#^	[39]
Acyl-CoA long-chain synthetase 1	*acsl1*	CGACCAAGCCGCTATCTC	CCAACAGCCTCCACATCC	55.0	97.8 ^#^	[13]
Acyl-CoA oxidase 1 3I	*acox1-3I*	TGTAACAAGGAGCAGTTCG	TTGCCGTGGTTTCAAGCC	56.0	96.9 *	[40]
Acyl-CoA oxidase 3	*acox3*	GGGAAGACGGCTACACACG	CAACAATTACTCCTGGCATCGC	55.0	105.3 *	[40]
Apolipoprotein AI	*apoa1*	ATGAAATTCCTGGCTCTTG	TACTCTTTGAACTCTGTGTC	55.0	89.9 ^#^	[41]
Estrogen receptor alpha	*erα*	GACATGCTCCTGGCCACTGT	TGGCTTTGAGGCACACAAAC	61.6	91.2 ^#^	[42]
Fatty acid binding protein 1	*fabp1*	GTCCGTCACCAACTCCTTC	GCGTCTCAACCATCTCTCC	57.0	97.7 ^#^	[41]
Fatty acid synthase	*fas*	ACCGCCAAGCTCAGTGTGC	CAGGCCCCAAAGGAGTAGC	60.0	95.1 ^#^	[43]
Glyceraldehyde-3-phosphate dehydrogenase	*gapdh*	CCACCTATGTAGTTGAGTC	ACCTTGAGGGAGTTATCG	55.0	92.8 ^#^ or 100.6 *	[41]
Hydroxy-3-methylglutaryl Co-A reductase	*hmgcoar*	CCTTCAGCCATGAACTGGAT	TCCTGTCCACAGGCAATGTA	58.0	94.2 ^#^	[43]
Lipoprotein lipase	*lpl*	TGCTGGTAGCGGAGAAAGACAT	CTGACCACCAGGAAGACACCAT	60.0	104.1 ^#^	[44]
Peroxisome proliferator-activated receptor alpha	*pparα*	CGGGTGACAGGGAGGTGGAGGAC	GGTGAGGATGGTGCGGGCTTTGG	59.0	100.6 ^#^	[45]
Peroxisome proliferator-activated receptor alpha Ba	*pparαba*	ATCCACTACTCCCACAGG	GTCTAAACCCAGCCAAATAC	55.0	106.7 ^#^	[41]
Peroxisome proliferator-activated receptor alpha Bb	*pparαbb*	GAGTCTCCTGTCCTATCC	AGTTCTGCTGTTCTTCAC	55.0	99.3 ^#^	[41]
Peroxisome proliferator-activated receptor gamma	*pparγ*	CGGAATAAGTGCCAGTAC	GGGTCCACATCCATAAAC	56.0	98.1 *	[46]
Ribosomal protein L8	*rpl8*	TCAGCTGAGCTTTCTTGCCAC	AGGACTGAGCTGTTCATTGCG	59.0	93.8 ^#^ or 99.0 *	[42]
Steroidogenic acute regulatory protein	*star*	AGGATGGATGGACCACTGAG	GTCTCCCATCTGCTCCATGT	63.0	104.5 ^#^	[47]
Vitellogenin A	*vtga*	AACGGTGCTGAATGTCCATAG	ATTGAGATCCTTGCTCTTGGTC	62.9	99.0 ^#^	[42]

# Amplification efficiencies determined with iQ SYBR Green Supermix. * Amplification efficiencies determined with SsoFastTM EvaGreen Supermix.

**Table 2:** Juvenile brown trout biometric data from the control (C), solvent control (SC), 17α-ethinylestradiol (EE2), atorvastatin (ATV), and mixture (MIX) groups.

Group	Total Weight (g)	Total Length (cm)	Fulton’s Condition Factor (K)	Liver Weight (g)	Hepatosomatic Index (HSI)
C	63.3 (41.3–83.3)	18.0 (15.3–19.5)	1.1 (1.0–1.3)	0.91 (0.66–1.20) ^b^	1.44 (1.28–1.60) ^c^
SC	62.3 (40.7–91.3)	17.4 (15.5–20.0)	1.1 (0.9–1.3)	0.93 (0.53–1.06) ^b^	1.37 (1.03–1.78) ^c^
EE2	62.8 (48.0–114.2)	18.3 (16.5–21.5)	1.0 (0.9–1.3)	2.13 (1.47–3.54) ^a^	3.30 (2.62–3.97) ^a^
ATV	52.8 (27.4–60.7)	17.0 (14.0–18.0)	1.0 (1.0–1.2)	0.73 (0.34–0.99) ^b^	1.37 (1.03–1.83) ^c^
MIX	48.2 (36.8–74.7)	16.5 (15.2–19.5)	1.1 (0.9–1.1)	0.87 (0.61–1.44) ^b^	1.84 (1.30–2.43) ^b^

Data expressed as median and (minimum–maximum) values for each group (*n* = 9 animals/group). For each biometric parameter, different lower-case letters indicate significant differences (*p* < 0.05) between groups.

**Table 3:** Juvenile brown trout blood lipid levels from the control (C), solvent control (SC), 17α-ethinylestradiol (EE2), atorvastatin (ATV), and mixture (MIX) groups.

Blood Lipid Levels										
Group	Total Cholesterol (mg/dL)	HDL (mg/dL)	LDL (mg/dL)	VLDL (mg/dL)	Non-HDL (mg/dL)	TGLs (mg/dL)	Total Cholesterol/HDL	HDL/LDL	LDL/HDL	TGLs/HDL
C	307.0 ^a^ (238.0–501.0)	105.0 ^a^ (101.0–109.0)	159.8 ^a^ (81.8–347.0)	48.0 ^b,c^ (43.2–59.8)	203.0 ^a^ (130.0–395.0)	240.0 ^b,c^ (216.0–299.0)	3.0 ^b^ (2.2–4.7)	0.7 ^b^ (0.3–1.3)	1.5 ^b^ (0.8–3.3)	2.2 ^b^ (2.1–3.0)
SC	376.0 ^a^ (247.0–434.0)	105.0 ^a^ (101.0–110.0)	215.2 ^a^ (92.2–281.8)	50.2 ^b^ (37.4–68.4)	269.0 ^a^ (138.0–332.0)	251.0 ^b^ (187.0–342.0)	3.5 ^b^ (2.3–4.3)	0.5 ^b^ (0.4–1.2)	2.1 ^b^ (0.9–2.8)	2.5 ^a,b^ (1.8–3.4)
EE2	421.0 ^a^ (192.0–501.0)	37.5 ^c^ (23.0–54.0)	138.0 ^a^ (109.0–280.0)	130.2 ^a^ (48.8–130.2)	198.0 ^a^ (169.0–374.0)	651.0 ^a^ (244.0–651.0)	8.0 ^a^ (4.9–9.0)	0.2 ^c^ (0.2–0.3)	4.5 ^a^ (2.9–6.0)	10.7 ^a^ (4.5–14.2)
ATV	151.0 ^b^ (107.0–235.0)	101.0 ^b^ (73.0–101.0)	44.0 ^b^ (3.4–89.8)	36.2 ^c^ (15.2–49.0)	66.0 ^b^ (24.0–134.0)	181.0 ^c^ (76.0–245.0)	1.9 ^c^ (1.3–2.3)	1.7 ^a^ (1.1–29.7)	0.6 ^c^ (0.0–0.9)	1.9 ^b,c^ (0.9–2.4)
MIX	335.0 ^a^ (269.0–501.0)	93.0 ^b^ (75.0–101.0)	196.0 ^a^ (130.0–350.0)	52.6 ^b^ (29.0–64.4)	234.0 ^a^ (193.0–400.0)	263.0 ^b^ (145.0–322.0)	3.6 ^b^ (3.3–5.0)	0.5 ^b^ (0.3–0.6)	2.0 ^b^ (1.6–3.5)	1.2 ^c^ (0.7–2.5)

Data expressed as median and (minimum–maximum) values for each group (*n* = 9 animals/group). For each lipid parameter, different lower-case letters indicate significant differences (*p* < 0.05) between groups. HDL—high-density lipoproteins; LDL—low-density lipoproteins; VLDL—very-low-density lipoproteins; TGLs—triglycerides.

## Data Availability

Dataset available on reasonable request from the authors.

## Supplementary Materials

The following supporting information can be downloaded at: https://www.mdpi.com/article/10.3390/toxics12030219/s1, Figure S1. Juvenile brown trout weight (n = 9 fish/group) in the different experimental groups (C—control, SC—solvent Control, EE2—17α-ethinylestradiol, ATV—atorvastatin and MIX—mixture) at the distinct injection days.
