# Supplementary material for: A Proof-of-Concept for a Hypolipidemic Brown Trout Model
Source: Toxics. 2024 Mar 15;12(3):219. doi: 10.3390/toxics12030219 (PMC10974606; doi:10.3390/toxics12030219)
Supplement: Supplementary file 1 [file toxics-12-00219-s001.zip › toxics-2890483-supplementary.pdf]

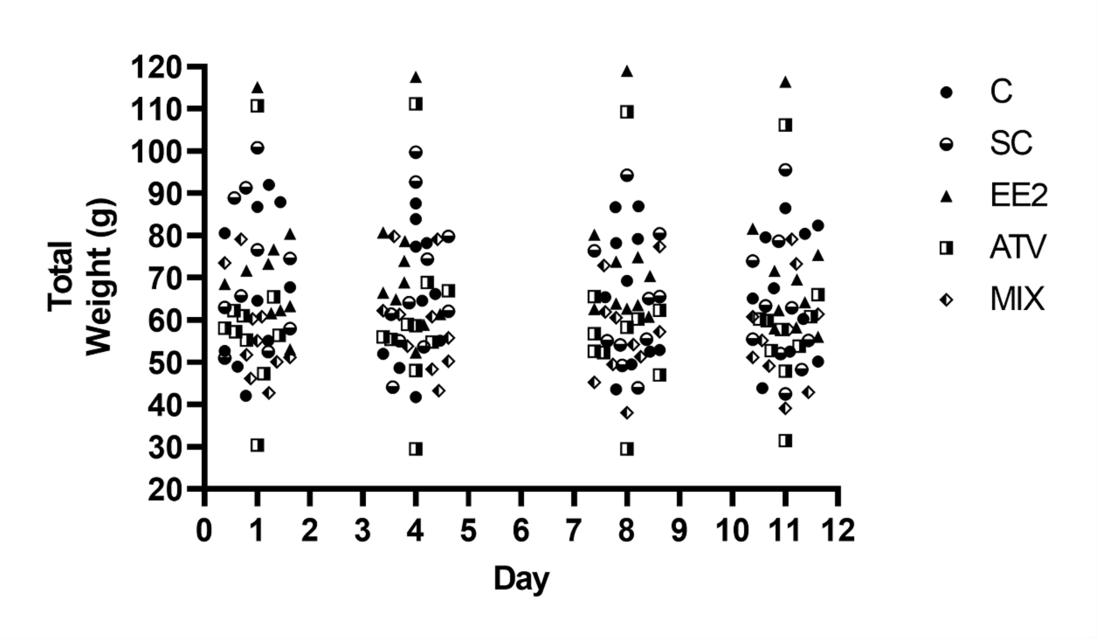

Figure S1. Juvenile brown trout weight (*n*= 9 fish/ group) in the different experimental groups (C – control, SC – solvent Control, EE2 – 17α-ethinylestradiol, ATV – atorvastatin and MIX – mixture) at the distinct injection days.
